# Roma Women’s Role in the Health Preservation of Their Communities during the COVID-19 Pandemic

**DOI:** 10.3390/ijerph21080985

**Published:** 2024-07-27

**Authors:** Paula Abella, Adriana Aubert, María Virginia Matulič, Ariadna Munté-Pascual

**Affiliations:** 1 Department of Social Work and Social Policies, University of Barcelona, 08035 Barcelona, Spain; paulaabella@ub.edu (P.A.); mmatulic@ub.edu (M.V.M.); 2 Department of Sociology, University of Barcelona, 08034 Barcelona, Spain; adriana.aubert@ub.edu

**Keywords:** Roma women, transformation, health crisis

## Abstract

The scientific literature has evidenced the stereotypes that affect the Roma people, which are detrimental to their access to the health systems in various countries. With the COVID-19 pandemic, this situation has been aggravated by falsely blaming, on many occasions, the Roma people as spreaders of the virus for supposedly not complying with the norms established by the health authorities. However, it has not been explored in depth what actions have been carried out by the Roma people during the pandemic to cope with this aspect. The aim of this article is to learn about the leadership of Roma women in relation to maintaining the health of their community during the pandemic. The research has been conducted through focus groups and life stories with a total of 47 Roma women and 24 Roma men participants, as well as interviews with 40 professionals from education, social services, health services, and civic organizations. The results show how the reality of the studied contexts was different to those stereotypes, that the Roma women in the contexts studied have led actions that preserved the health of their communities, that the established measures were strictly followed, breaking with the extended stereotype about the Roma people.

## 1. Introduction

The impact of geography and social group membership on health is not only powerful but also persistent. Health inequalities across social groups can be generated early or late in life by differences in access to material resources and social circumstances that generate stress or health behaviors [1]. Such health disparities are of particular concern among vulnerable and socially excluded groups such as Roma people [2].

According to World Health Organization (WHO) estimates, between 10 and 12 million Roma live in the European Region, of which 6 million are estimated to live in the European Union alone, and this community is disproportionately poor in many countries, with data indicating that they are concentrated among the poorest. Moreover, the conditions in which most Roma people live have serious health consequences [3]. Furthermore, Roma are highly affected by communicable diseases and due to the cultural blindness of health institutions, they face barriers to participating in and navigating the health system [4,5]. The Council of Europe reports that the life expectancy of Roma and Travelers is, on average, ten years lower than the general population due to pre-existing risks, such as hunger and malnutrition, lack of vaccination, precarious housing, and significantly higher unemployment rates [6]. The impact of the gender variable in the field of health is also relevant, as Roma women are vulnerable in the first place because they are women, because they belong to an ethnic minority, and because they have faced discriminatory treatment in health, school, and social services [7,8].

Secondly, estimates based on data collected in 2021 by the European Union Agency for Fundamental Rights suggest that Roma women live on average 11 years less than women in the general population, and for an average of 71.3 years compared to 82.2 years for non-Roma women [9]. At the same time, it is worth mentioning that when analyzing gender differences in EU member states, more Roma and non-Roma women than men report that their daily activities are limited by health problems [10].

In terms of the national framework, the Roma community in Spain has a worse health status than the socio-economic groups better placed in the occupational hierarchy [11] and the prevalence of chronic diseases among this population is high [12]. During the period covered by the National Health Surveys of the Roma Population, carried out in 2006 and 2014 by the Ministry of Health, Social Services and Equality in Spain, there was no reduction in health inequality concerning the general population, nor was there a reduction in the gender gap in health for either population [13].

Taking into account the evidence collected so far, the situation of Roma women in the field of health is important, not only because they have different levels of structural vulnerability than their male counterparts and women in the majority population, but also because of their leading role as the ones responsible for transmitting culture and values and as an essential part of family self-care [14]. Concerning the above evidence on the scale of values and Roma culture, it is worth mentioning that two cultural elements of this community play a key role in their state of health: family cohesion and mutual care and protection, so the feeling of common identity motivates the strong bonds of solidarity that they develop among themselves, a factor that can contribute to improving their health [2]. At this point, and taking into account the previous contributions on the cultural role of Roma women, it is worth highlighting that recent scientific evidence shows that Roma women are the driving force for change thanks to their leadership capacity within their families and communities [15,16]. At the same time, they are the creators of solidarity networks that improve their access to basic services such as health and social services and also present a greater permeability towards changes specifically related to the role of health [16,17].

Looking more closely at the health of Roma women, they report a higher prevalence of indicators such as high blood pressure, osteoarthritis, asthma, diabetes, cholesterol, depression, mental health problems, migraine, and problems related to menopause [18]. It is also worth mentioning the incidence of certain diseases derived from the role of mothers and wives: high fertility rate with pregnancies and births at very early and even advanced ages, poor information regarding family planning (lack of knowledge of contraceptive methods and persistence of myths), poor prevention of gynecological diseases, premature ageing, with incidence of diseases that are not typical of age (diabetes, bone conditions, cardiovascular problems), and excessive responsibility inside and outside the home, with the consequent appearance of symptoms of depression, anguish, and anxiety in some cases [19].

Considering the health inequalities of both men and women in the Roma community compared to other ethnicities, we cannot ignore the entry of SARS-CoV-2 (COVID-19) disease, as the Covid crisis has had many implications for population health worldwide and has drastically affected vulnerable groups in society [20,21], especially the Roma community [9]. Likewise, marginalized groups are at increased risk of pre-existing conditions that increase the risk of COVID-19 infection (including death), such as high rates of underlying chronic diseases that can directly or indirectly worsen Covid outcomes, respiratory diseases, HIV, viral hepatitis, tuberculosis, diabetes, cancer, cardiovascular and mental illness, along with substantial barriers to testing, care, and information about Covid [22].

The arrival of the COVID-19 pandemic disrupted the ‘normal’ functioning of societies by affecting people’s ability to socialize, work, receive education, access health care, and maintain an income, and these effects were more pronounced for certain groups due to their pre-existing precariousness, poverty, and lack of capital [21,23,24]. On the other hand, one study finds that during the pandemic, in some countries, the political discourse of security elites constantly portrayed Roma as a threat and the majority as those to be protected [25]. Anti-Gypsyist sentiments and discourses have also exacerbated adverse effects, and Roma have become victims of racist aggression, collective victimization, and ethnic scapegoating [26]. In addition to the difficulties experienced by the Roma community in the context of the pandemic and health crisis, it has been found that public health measures often led to a decrease in income, as closures and distancing measures hindered people’s ability to work and entailed certain costs (masks, disinfectants, etc.) [23].

Another added difficulty has been in the economic sphere, as the main economic activities of many Roma are related to traditional occupations such as trade, production, and sale of handicrafts and metalwork [26]; for many people who often work in the informal sector, these measures posed obstacles to affording food and paying utility bills. Paying for masks and disinfectant was an additional cost that some could not afford [23]. On the other hand, enforcing social distance within these communities can be difficult, as many do not have the space to do so [27]. Existing spatial deprivation, poor housing, and inadequate access to infrastructure limited the extent to which Roma could comply with hygiene rules, including social isolation or confinement [23]. Thus, social determinants also contribute to making people in marginalized communities more vulnerable to COVID-19 infection, even when they do not have underlying diseases [28].

This article aims to provide information on the impact of Covid on the health of the Roma population, focusing on the leadership capacity of Roma women, as well as on the protective factors that facilitate the maintenance of a good state of health in a situation of adversity.

## 2. Materials and Methods

The research presented stems from the RTD project Evidence-based solutions for the overcoming of COVID-19 effects on the Roma community, Roma women leading communities’ transformation (ROM21) funded by the Spanish Ministry of Science and Innovation (MCIN) and the State Research Agency (AEI). This is a qualitative study that follows the communicative methodological orientation [29], which guarantees the inclusion of the voices of the participants in the study throughout the entire methodological process. The main techniques used in this exploratory study were (a) a review of the scientific literature obtained from the main scientific databases, such as WOS, JCR, and SCOPUS; (b) in-depth interviews with professionals from social services, medical institutions, educational centers, and non-profit organizations (40 professionals in total); and (c) communicative accounts and discussion groups of men and women and Roma families (47 women/24 men).

The communicative methodology has been a pioneer in the current criteria for all sciences in the main research programs, such as the European Commission’s [30] social impact and co-creation. This means an orientation of research and its results to achieving societal goals [31], and that knowledge is constructed in an egalitarian dialogue between researchers and participants [32]. It is worth mentioning that the research that took these two criteria to the European level for the first time was precisely co-created with Roma people, which obtained, as a result, the recognition of the Roma people as a European minority by unanimity in the European Parliament in 2005, and later, other state members did so too.

Concretely, researchers contribute to the dialogue through scientific knowledge, and the participants contribute through their lifeworld’s knowledge. In all the fieldwork carried out, the objective was to gather information on the actions led by Roma women in order to overcome the difficulties posed by the pandemic. Concretely, the focus was on the following Sustainable Development Goals: gender equality, no poverty, quality education, decent work, and reduced inequalities. Therefore, all the dialogues in the fieldwork revolved around the mentioned five goals and these three fields: education, social services, and civic organizations.

It should be noted that our research group, unlike the internal resources available in the hospitals that make up the Spanish health system, has not had access to ethnicity registers that provide information on the number of people of Roma ethnicity who have been infected by the SARS-CoV-2 virus (COVID-19). It is therefore essential to mention that both ROM21 and this article work with the data provided by the testimonies of the different agents involved, starting at the same time from the stories told by the Roma women and the narratives of the professionals who provided care in a situation of adversity and health urgency.

Data collection was carried out in three Spanish regions (Catalonia, Basque Country, and Aragon) during the first two years of the project (2022–2023). Most of the interviews and focus groups with Roma were conducted face-to-face in natural spaces that allowed participants to feel comfortable interacting with the research team. However, online interviews were also conducted via WhatsApp or other platforms such as TEAMS or Zoom [33,34]. In the case of professionals, the remote option was the most prevalent. The data obtained in the fieldwork have been transcribed and coded for the data to be analyzed through the analytical categories established for this purpose [35,36].

At the ethical level, the research has proceeded according to the deontological precepts that appear in the Helsinki [37] and Taipei [38] Declarations of the World Medical Association. All collaborators have been informed about the ROM21 project, the treatment of their data, and the subsequent publication of results through reports, articles, and congresses, and have signed an informed consent form for the record. The research has also received ethical approval from the Community of Research on Excellence for All (reference number 20230212).

The aim of this article derived from the results of the ROM21 (2021–2024) Project is to provide knowledge on the impact of the pandemic on the health of Roma families, overcoming negative stereotypes by making visible the leadership role of Roma women in maintaining family health during the pandemic and post-pandemic context. With this objective in mind, the following analytical categories have been described in Table 1:

In accordance with the communicative methodology, each of the defined analytical categories is analyzed according to its own double dimension of exclusion (negative elements) or transformation (positive elements, which overcome the barriers experienced by the Roma). On the basis of the above, the resulting analytical table is shown below in Table 2:

It should be noted that the underlying perspective of the ROM21 project assumes that the category of female leadership (C5LF) is transversal, so that each category and its consequent dimensions intersect with the aforementioned category C5LF, as can be seen in the presentation of the results in point 3 of the results.

## 3. Results

The results obtained from the analysis of empirical and documentary data are structured on the basis of the categories described in the previous section in order to elucidate the impact of the SARS-CoV-2 virus (COVID-19) on the most important areas related to the health of the Roma population, always in intersection with the female leadership variable. Thus, this section is made up of the following six sub-sections: impact on physical health, impact on mental health, monitoring of prevention, protection and isolation measures, use of health resources and material, vaccination, and lastly, access to information during the pandemic. Although exclusionary elements have also been found, the focus of the research is on those actions and features that have enabled overcoming them. Following the scientific criteria of social impact [30], attention has been paid to those actions and elements that have improved the lives of Roma people during the pandemic in relation to the Sustainable Development Goals.

It must be highlighted that the interviewed Roma men mainly emphasized the role of Roma women as transmitters of essential information around the virus and how to protect themselves from it. This is why most of the examples chosen to illustrate this issue in this article are only quotations made by Roma women, complemented by professionals, especially social workers, given that the research is focused on Roma women’s social action in relation with these professionals. Roma men, in our research, did not say they did not take part in this matter; however, they specified that Roma women had a key role in the transmission of information and behaviors in their communities and families. HVHA1, a Roma man, put it this way:

I believe that the Roma woman is justly distinguished or identified because she is the one who is the mover inside the home, the one who spreads values in one way or another, she is the one who is the center in terms of morality and values. So, in a way that I think is logical, all this that we are talking about has also fallen on her, hasn’t it? (HVHA1, Roma man).

### 3.1. Incidence on Physical Health

Participants stated that many Roma people had some health issues that were aggravated, but that many solidarity actions led mainly by Roma women were able to reverse or alleviate these situations. As an example, in relation to food and the ability to exert a positive influence through the female leadership of Roma women, it should be emphasized that during the social emergency resulting from the pandemic, one of those interviewed in our study, together with other members of the community, led an initiative to alleviate the effects of food poverty by promoting a food bank. One of the women interviewed in our study, together with other members of the community, led an initiative to alleviate the effects of food poverty by promoting a food bank to provide care for different groups in her neighborhood:

There’s a bit of everything, but no, because my neighborhood is colorful, and well, the neighborhood was a bit lonely, but it was the Roma community, the Roma women who took the lead, and so now I speak as [her name], I was the promoter of a food bank […] and well, in the end I gave to everyone, it was for my people. And the Roma women went to the food bank, but not just the Roma women, the Nigerian woman, the Arab woman, the woman from the majority community, everything was full of women (HVMC4, Roma woman).

On the other hand, as one of the social workers interviewed in the area of Basic Social Services recalls, Roma women also developed the role of intermediary with the Public Administration about the satisfaction of basic needs and the demand for food, adopting an active role in the resolution of the different problems arising from a context of adversity.

And then when there was an outbreak of Covid in [name of the place] and all that, [name] went to the [center for social emergencies and emergencies], they activated the home visits. […] They went here, to tell her but they had already done all the work, because she had gone to emergencies and they said: “Don’t worry, we’ll bring the food” (ESCOCPC 6_2, social worker).

### 3.2. Incidence on Mental Health

Another of the main effects of the pandemic has been in the area of mental health. The results obtained show that, throughout the different stages of the pandemic, the psychological and emotional well-being of Roma women has been affected by different circumstances. Some of these circumstances have come from the pandemic context itself, the situation of ignorance and insecurity, as well as the impossibility of maintaining face-to-face contact with members of their extended family or social environment in certain moments of adversity. At this point, it is worth recalling the importance of the scale of values of Roma culture, including the close social and emotional ties they maintain with their families, and how the pandemic context has affected this circumstance.

It has affected me above all in terms of insecurity and mistrust, because it was something we didn’t know what it was like, a bit of uncertainty and a bit of psychosis because we didn’t know what it was all about, so the truth is that I was always in front and very strong in front of my children because there was nothing else left […] and we had nothing else left but fear. Fear, but not showing it to my children, fear and insecurities’ (HVMA3, Roma woman).

Yes, but I think it is more nostalgia than anything else, but not being able to accompany a person in hospitals or cemeteries or whatever ails them, I think it has left us all touched (HVMA1, Roma woman).

With regard to the female leadership of Roma women in the field of mental health, it is worth highlighting the strategies undertaken at various times during the pandemic aimed at alleviating the effects of the health crisis on their families, especially with children at home, highlighting the relevance of their role as mothers and carers. The following contributions are an example:

They were very small of course, and I didn’t want it to affect them when we came back to life and they were afraid to relate to people, I tried by all means all the time to keep them active, we created a blackboard, we did activities, then upstairs or in the block is the terrace and my husband occasionally took them upstairs and there with them played, gave them air, the babies were there for a while and it was the way they went out somewhere (HVMC6, Roma woman).

I remember that my older girl had a kind of anxiety attack, she started to cry a lot, she wanted to go out, she wanted to go outside, I just remember that I was sitting in the shower with her waiting for her to get over the anxiety attack she had, I remember that I put her pajama’s on, I was with her next to the bed until she got over the attack (HVMA2, Roma woman).

### 3.3. Follow-Up of the Measures for the Prevention, Protection, and Monitoring of COVID-19

By looking more closely at the factor of fear as a determining factor, and in contrast to the prejudices and stereotypes that have emerged at various levels of the collective imagination, which blur the image of the Roma community as being responsible for the spread of the virus, the results analyzed in our research determine a broad follow-up of the prevention, confinement, and social isolation measures dictated by the relevant public health administrations for this community, corroborated by both the professionals and the women interviewed. One of the reasons why this follow-up of the measures was more extensive with the target population of our study was related to the value of the family and mutual protection among the members of the same group in a situation of vulnerability according to the analyzed contexts.

At the Roma level we had very few because the families always tried to manage somehow to take care of the sick person at home, if they had to isolate them they would isolate them’ (ESOC1, social worker).

Because when I caught it, the truth is that when I was discharged I could leave the room, I didn’t leave the room directly, in other words, I stayed there for four more days because I wanted to. And of course I had to leave the room with gloves, with disinfectant, I sat on the sofa and put a plastic bag […] I took the sheets off every day, I washed the clothes at sixty, with bleach, we ate with plastic things to throw away, I didn’t want anyone in the house, it was an obsession, I didn’t want to go out in the street. (HVMC10, Roma woman).

At the same time, from the point of view of the health professionals who provided direct care throughout the pandemic, it is worth noting that there was a high demand for antigenic tests to detect infection among Roma people.

During the first few months of the pandemic, because no one was to be seen on the street, so it was very strict (…) When the old tests came out, the ones that were done nasally, that were done at the center, they came repeatedly, the whole family came, even when we were told that they could not be done indiscriminately and the primary health center did not do it indiscriminately, but with a selection criteria, because they came to us at night to do it to the whole family and we did it because you could see how anxious they were, and maybe they came two, three or four times a month to do it, when someone thought they might be infected, they would come and if they could, they would take the whole family (EOSC2, health professional).

The fact that the Roma community, particularly in the case of women, has exhaustively followed the health and hygiene indications, measures, and recommendations is in itself a protective factor in the area of health in an adverse situation such as COVID-19, as it has contributed to reducing the chances of developing other types of infections and illnesses that are characteristic of the pandemic context itself, such as respiratory infections and allergies:

Yes that maybe there have been as it seemed that they were so scrupulous with the cleanliness and so, maybe other pathologies have not had them as they could be respiratory infections and so, there have been fewer, but there have also been fewer in general, and also in terms of allergies, it has been seen that there have also been fewer cases due to wearing masks and that there have been fewer outings’ (EOSC2, health professional).

At the same time, in this section, it is worth highlighting the importance of the role of the Roma woman in the family and specifically in making decisions that influence the health and monitoring of protection measures in her immediate environment:

Yes, the Roma woman has done a lot, because yes, they have been the ones who have taken care of the children, they have taken the children forward, taken them to the doctor, taken the family forward (HVMA4, Roma woman).

### 3.4. Utilization of Health Resources and Equipment

With the above, it is worth briefly mentioning another effect of the exhaustive monitoring of the protection and prevention measures imposed by the health authorities on the Roma community, namely the decrease in attendance at health centers during the first months of the pandemic. However, once the initial impact had passed, and with the corresponding evolution of the health crisis, which led to a relaxation of the first measures adopted to prevent the spread of the virus, the levels of attendance of the Roma population at health centers began to recover:

The first month, when there was a stricter confinement, attendance dropped a lot. In other words, the Roma families were very strict with the confinement, there was a lot of fear […] The women had more fear than the men […] It was fear […] It was a fear […] I believe that the women had more fear than the men […]. It was a fear, I think that maybe they had a lot of fear for their children, for their families or for themselves, but there was a lot of fear […] Attendance went down a lot […] Afterwards, as it became more relaxed, they started to come (EOSC2, health professional).

Nonetheless, and following on from what is explained in previous lines, it is worth noting that at certain times, the Roma women, through group cohesion and networking, managed actions aimed at providing health and protection material against the SARS-CoV-2 virus (COVID-19) in some areas of the city of Barcelona for people who needed it, once again demonstrating their ability to have a positive influence on the community:

And with the sewing workshop that they facilitated, we even started sewing masks. People who didn’t have any resources to protect themselves contacted us through a Facebook page and told us ‘We are so many people, we need so many masks’. […] I was super pregnant, with my belly, I think I sewed like 100 or 200 masks, and it was my way of being able to help (HVMC13, Roma woman).

### 3.5. Vaccination and Information

Before delving into the communication stories of Roma women in relation to vaccination and information from COVID-19, it is worth mentioning first of all the role played by the organizations that work with the Roma community in two areas. On the one hand, they articulated different intervention strategies to meet the needs identified on the basis of the interpretation of the agents involved, especially in the field of health, and on the other hand, they promoted access to reliable information on different aspects related to the pandemic context.

Secondly, at the same time, it has been possible to confirm that the information, awareness-raising, and networking work carried out by the organizations that provide care to the Roma population has also proved to be a key protective factor in promoting a very broad follow-up of the vaccination guidelines in the population that was the subject of our study:

By complying so much with the norms, and the confinement, they came to ask for medication, as soon as the vaccine came out, they all wanted to be vaccinated immediately. (EOSC2, health professional)

Here there has been a lot of campaigning, at least there have been awareness campaigns and there was also a group of certain associations like [names], there were a series of entities that joined the vaccination, and the truth is that people here have been very receptive, and many people have gone to get vaccinated, there are others who haven’t. There is a percentage who are very afraid, but there is another percentage who want to get vaccinated and so they can go out on the street and they have more freedom. And here they would say to me ‘But I can’t find an appointment, but who do I have to call?’ And the truth is that I can’t complain that there are families who haven’t wanted to get vaccinated, I can say that the vast majority of the Roma population here, for example, in my case, people have been very receptive and have been vaccinated. (ESOCPC3, social worker)

Thirdly, and once again, it has been possible to confirm the positive influence that Roma women have on their community and on decision making in the health field, in some cases contributing to the promotion of vaccination among their peers:

Well, here in the neighborhood they have been vaccinated quite a lot, there are those who didn’t want to, but those who are at risk, those who have children at risk and that’s why they ask me: ‘Well, what do you say about the vaccine?’ And I said: ‘I’ll get vaccinated, I want to hug my mother’. Of course, when you talk about you to them, they see that you have the same problems as them and that you are there, so yes, here they have been vaccinated a lot (HVMC5, Roma woman).

Next, in Table 3, there is a summary of the participants in our research in which more than half of the women, 53%, have had the COVID-19 vaccine.

## 4. Discussion

The present results are in line with previous evidence that shows the human agency capacity of Roma women [5,39]. The testimonies by the women themselves and professionals uncover that, in the studied contexts, Roma women have employed diverse strategies to alleviate the negative effects of the pandemic, both in formal contexts in the public sphere and informal contexts of the private sphere and the community setting.

Taking into account the conceptualization of leadership as a generative process of influence of effects on the community, which implies a compromise relationship between the leader and the community [40], we highlight, in the first place, the leadership in alleviating food poverty and the gathering of sanitary material and of protection needed to face the pandemic. Regarding food, we can find testimonies such as the one by HVMC4 that referred to the creation of a food bank as a spontaneous reaction of a group of Roma women in the face of the situations with bare necessities that they had observed, going beyond ethnic frontiers; this fact reflects the effectiveness when it comes to covering such a basic need. This circumstance develops as a protective factor that, in their settings, goes in line with recent contributions from scientific literature that show how Roma women, far from being mere spectators of their social reality, become agents of social transformation [41] through a significant exercise of individual and collective responsibility (derived from their role inside the family units) that has positive influence in their community setting.

In relation to health, our testimonies show how, throughout diverse periods of the pandemic, Roma women participating in this study have led strategies to preserve their own mental health and those of their family. On the other hand, identity characteristics of the Roma culture and their value scale have enabled them to keep contact and the affective relations with their social contacts in a context of social isolation. This contribution adds to the body of specialized scientific literature that shows how specific characteristics of Roma culture are a key factor in crisis management [42], which can be applied to the health crisis experienced in recent years.

With this in mind, and taking into account the trajectories exposed by the interviewed Roma women, their voices and intersectionality remain patient in their role as women, mothers, and caregivers; the protagonists of this study have evidenced distinctive features that have enabled a transformative role in the health area: to lead, to organize, to influence, and to implement a great part of the protective factors and enablers of a good state of health in their communities, as has been seen in the previous section.

Parallel to the implications of Roma women, it is of great relevance to collect the synergies that they have maintained with agents from the public health system and other health services, evidencing the effort the latter has made in the analyzed contexts. These agents have worked with the Roma people, trying to reach the maximum population possible in a situation of health and social emergency; they have articulated a response to the necessities the Roma people were presenting in this particular context. Moreover, it has been evidenced that, to achieve an integral response to a health problem that has been the spread and the evolution of the COVID-19 virus, following the scientific community’s contributions, public health strategies foresee a strong collaboration among public institutions, social organizations, and the community of a specific territory [43].

In the studied contexts, it is precisely following the action of diverse agents, both from public administration and from the community, where there have been actions fostering the participation and the direct contributions of Roma people in the decision making in the health field, initiatives that, in turn, contribute to the overcoming of power dynamics that cause their exclusion [44]. The access to accurate information and the levels of vaccination in the studied contexts through the prevention and sensibilization of entities that work with Roma people are an example of this cooperation. This relationship has been between the stakeholders of a social reality where the lines of action arise from the necessities of the same agents.

## 5. Conclusions

The present article is part of the RTD project “Evidence-based solutions for the overcoming of COVID-19 effects on the Roma Community. Roma women leading communities transformation (ROM21)”, which contributes to the international scientific debate about how, in the studied contexts, the Roma, as a transnational community, have their own mechanisms and potentialities that are to be taken into account in order to improve their living situations, being that Roma women are key leaders of such transformations [45,46]. Scientific evidence focused on the protective factors established by the very agents that are part of a community in an adverse situation of crisis is needed, and our article contributes to creating scientific knowledge about this new methodological perspective. At the same time, the results obtained in this research contribute to the overcoming of prejudice and stereotypes traditionally maintained towards the Roma, by recognizing their diversity. Equality of differences implies the right to be different and to be equally valued, which is why it is key to guarantee dialogic processes that include the voices of the Roma population, as well as respect and reciprocal knowledge [42].

## Figures and Tables

**Table 1:** Analytical categories related to the impact of the pandemic on health and Roma women’s leadership in overcoming the impact.

Code	Name	Description
C1SF	Physical Health	Covid, Covid-derived disease, chronic disease, common illness, nutrition, physical activity
C2SM	Mental Health	Depression, anxiety, abulia, other severe mental disorders
C3RSS	Social and health resources	Psychosocial care, medical care, food, hygiene products, money, sanitary products, medication
C4I	Information	Media, community networks, social organizations, health and educational institutions
C5V	Vaccination	Attitudes towards vaccination, level of knowledge, vaccinated people
C6LF	Women’s leadership	Social actions towards the community, caregiving role, family organization, decision making, maintenance role, family dynamics

Source: Own elaboration based on the ROM21 Project.

**Table 2:** Table of analysis.

Category	Exclusionary Dimension	Transformative Dimension
C1SF	1	2
C2SM	3	4
C3RSS	5	6
C4I	7	8
C5V	9	10
C5LF	11	12

Source: own elaboration based on the ROM21 Project.

**Table 3:** Compilation of the interviewees in the research who have had the COVID-19 and who have been vaccinated.

Interviewees	COVID-19	Vaccine
HVMC1	Without information	Yes
HVMC2	Yes	Yes
HVMC3	Yes	Yes
HVMC5	Without information	Yes
HVMC6	No COVID-19	Yes
HVMC7	Yes	No
HVMC8	No	Yes
HVMC9	No	Without information
HVMC10	Yes	Yes
HVMC11	No	Yes
HVMC12	No	Yes
HVMC13	Without information	Without information
HVMC14	No	No
HVMC15	Yes	Yes
HVMC16	No	Yes
HVMC17	Yes	No
HVMC 18	Yes	Yes
HVMC19	Yes	Yes
HVMA1	Yes	Without information
HVMA2	Without information	Without information
HVMA3	Without information	Without information
HVME1	Without information	Without information
HVME2	No	Without information
HVME3	Yes	Without information
HVME4	No	Yes
HVME5	No	Without information

## Data Availability

Data are available upon reasonable request to the corresponding author.
